# Hospital indicators and inpatient behavior in a psychiatric hospital that implemented the smoking ban

**DOI:** 10.1590/1518-8345.5666.3548

**Published:** 2022-08-01

**Authors:** Renata Marques de Oliveira, Jair Lício Ferreira Santos, Antônia Regina Ferreira Furegato

**Affiliations:** 1 Universidade Federal de Minas Gerais, Escola de Enfermagem, Belo Horizonte, MG, Brasil.; 2 Universidade de São Paulo, Faculdade de Medicina de Ribeirão Preto, Ribeirão Preto, SP, Brasil.; 3 Universidade de São Paulo, Escola de Enfermagem de Ribeirão Preto, Centro Colaborador da OPAS/OMS para o Desenvolvimento da Pesquisa em Enfermagem, Ribeirão Preto, SP, Brasil.

**Keywords:** Smoke-Free Environments, Smoke-Free Policy, Indicadores de Servicios, Agression, Hospitals, Psychiatric, Psychiatric Nursing, Ambientes Livres de Fumo, Política Antifumo, Indicadores de Serviços, Agressão, Hospitais Psiquiátricos, Enfermagem Psiquiátrica, Ambientes Libres de Humo, Política para Fumadores, Indicators of Health Services, Agresión, Hospitales Psiquiátricos, Enfermería Psiquiátrica

## Abstract

**Objective:**

to compare hospitalization and discharge indicators, medication costs and patient behavior before and after the implementation of the smoking ban in a psychiatric hospital.

**Method:**

ecological, longitudinal and retrospective study carried out in a psychiatric hospital. Secondary data referring to 2142 hospitalizations were collected from medical records. The median test was used to compare the variables before and after the ban.

**Results:**

after the implementation of the ban, there was a reduction in bed occupancy rate in male units for mental disorders (from 88.8% to 48.4%) and substance dependence (from 94.4% to 42.8%). There was a reduction in the mean length of hospital stay in the male chemical dependency unit (from 13.5 to 12.6) compared to the female unit (from 14.7 to 19.5). There was a reduction in costs of psychotropic drugs and expectorants, episodes of verbal/physical aggressions and physical/chemical restraints.

**Conclusion:**

the smoking ban changed hospital indicators, reduced costs and improved patient behavior, contradicting the myth that it results in hostility. It is hoped that this study will help nurses to review their beliefs related to smoking cessation, as there were positive results for interpersonal relationships and for the management of mental health services.

## Resumo

**Objetivo:**

comparar indicadores de internação e de alta, custo com medicamentos e comportamentos dos pacientes antes e após a proibição do tabagismo em um hospital psiquiátrico.

**Método:**

estudo ecológico, longitudinal e retrospectivo realizado em um hospital psiquiátrico. Foram obtidos dados secundários por meio de consulta aos prontuários, referentes a 2142 internações. Aplicado teste de medianas para comparação das variáveis antes e após a proibição.

**Resultados:**

com a implementação da proibição, o percentual de ocupação dos leitos foi reduzido nas unidades masculinas de transtornos mentais (de 88,8% para 48,4%) e de dependência química (94,4% para 42,8%). A média de dias de internação foi reduzida na unidade masculina de dependência química (13,5 para 12,6) em comparação à unidade feminina (14,7 para 19,5). Os custos com psicofármacos e expectorantes, as agressões verbais/físicas e as contenções físicas/químicas foram reduzidos.

**Conclusão:**

a proibição do fumo alterou indicadores hospitalares, reduziu custos e melhorou o comportamento dos pacientes, contrariando o mito de que ela resulta em hostilidade. Espera-se que este estudo contribua para que os enfermeiros revejam suas crenças relacionadas à proibição do tabagismo com resultados positivos para as relações interpessoais e para a gestão dos serviços de saúde mental.

## Resumen

**Objetivo:**

comparar indicadores de internación, alta, costo con medicamentos y comportamientos de los pacientes antes y después de la prohibición del tabaquismo en un hospital psiquiátrico.

**Método:**

estudio ecológico, longitudinal y retrospectivo realizado en un hospital psiquiátrico. Fueron obtenidos datos secundarios, a partir de consulta a las fichas médicas, referentes a 2.142 internaciones. Fue aplicado el test de medianas para comparación de las variables antes y después de la prohibición.

**Resultados:**

con la implementación de la prohibición el porcentaje de ocupación de las camas fue reducido en las unidades masculinas de trastornos mentales (88,8% para 48,4%) y de dependencia química (94,4% para 42,8%). La media de días de internación fue reducida en la unidad masculina de dependencia química (13,5 para 12,6) en comparación con la unidad femenina (14,7 para 19,5). Los costos con psicofármacos y expectorantes, las agresiones verbales/físicas y las contenciones físicas/químicas fueron reducidas.

**Conclusión:**

la prohibición de fumar alteró los indicadores hospitalarios, redujo costos y mejoró el comportamiento de los pacientes, contrariando el mito de que esta resulta en hostilidad. Se espera que este estudio contribuya para que los enfermeros revisen sus creencias relacionadas con la prohibición del tabaquismo, considerando los resultados positivos para las relaciones interpersonales y para la administración de los servicios de salud mental, que fueron obtenidos.

**Highlights**

(1) The smoking ban changed hospital indicators in public units.

(2) The smoking ban resulted in reduced costs on psychotropic drugs and expectorants.

(3) The smoking ban resulted in a reduction in verbal and physical aggression.

(4) The smoking ban has positive results for the management of health services.

(5) The results contradict the myth that smoking bans increase aggression.

## Introduction

Smoking is a serious public health problem. Its prevalence is estimated to be 15% in the world population and 9.8% in Brazil^1^ ^-^ ^2^. In the psychiatric population, the situation is even more concerning: one-third of Americans with mental disorders smoke tobacco and, in Brazil, the prevalence varies from 25% to 60%, depending on the mental health service investigated^2^ ^-^ ^4^.

The implementation of Brazilian Law no. 12.546/2011, which prohibits smoking in collective environments (public and private), is a challenge for mental health services, especially psychiatric hospitals, which have a higher prevalence of smokers and treat people with more severe tobacco dependence. In addition, the culture of smoking (using cigarettes as currency), inherited from asylums, is still common in these places^4^ ^-^ ^5^.

As a result, there is still resistance to smoking bans in mental health services. Recent studies carried out in Iran and Italy show that professionals are skeptical about the successful implementation of a smoke-free policy in these places^5^ ^-^ ^6^. A Brazilian study revealed that the fear that the ban could affect hospital indicators, reducing the number of admissions and increasing discharge on request, for example, is one of the reasons for resistance in psychiatric hospitals, especially in private and philanthropic institutions^7^.

Scholars argue that the smoking ban during psychiatric hospitalization is an opportunity for people with mental disorders to rethink this habit, receive guidance on appropriate strategies for coping with and maintaining abstinence, have their degree of dependence evaluated, and adhere to therapeutic recommendations^8^.

Although it is a challenge, there is scientific evidence of the benefits of smoking cessation for people with mental disorders: increased quality of life; improvement of anxiety and depression; reduction of aggressive behavior; reduction of drug relapse rates; improvement of physical symptoms; and reduced risk of heart disease and cancer^9^.

Another potential benefit is the reduction of medication costs for smokers, considering both clinical and psychotropic drugs, as there is scientific evidence of improvement in psychiatric symptoms. A study carried out in the United Kingdom with 13,846 people with serious mental illness revealed that physical comorbidities and smoking were associated with higher costs with medication and diagnostic and professional tests^10^. Considering this evidence, for the psychiatric population, smoking cessation is an advantage both for those who are no longer exposed to the harmful effects of smoking and for managers and government agencies that deal with health financing, as smoking habits affect the process of service management.

Considering the indispensability of smoking cessation in the psychiatric population, the legal requirement for a smoke-free policy in mental health services, and the smoking ban in these places as an opportunity for psychiatric patients to rethink the habit, this study fills a current gap, as the theme is still not thoroughly investigated in the Brazilian context, which limits the access of healthcare professionals working in mental health services to the knowledge of experiences carried out in the country.

Based on the above, the present study aims to test the hypotheses: 1) The smoking ban affects hospital indicators; 2) The smoking ban reduces aggressive behavior; 3) The smoking ban reduces costs with psychotropic drugs and expectorants.

This study aimed to compare hospitalization and discharge indicators, medication costs and patient behaviors before and after the smoking ban in a psychiatric hospital.

## Method

### Type of study and period

Ecological, longitudinal, and retrospective study, carried out in 2020 with secondary data obtained from September 2017 to August 2018. The study is classified as ecological because exposure and outcome were obtained from aggregated data (hospital indicators that refer to a group of individuals). It is classified as longitudinal because it compares, over time, groups of individuals hospitalized, before and after the implementation of the smoking ban. Finally, the study is also retrospective, as the data obtained refers to a period prior to data collection (past data)^11^.

### Study setting

The study was carried out in a philanthropic psychiatric hospital with a private administration, located in the countryside of the state of São Paulo. It was chosen for convenience as, in March 2018, it adhered to Law 12,546/2011, which prohibits smoking in collective environments.

The hospital has 107 psychiatric beds for hospitalizations through the Unified Health System (SUS), 60 psychiatric beds in a private unit, 20 private beds in a daily regime, and 28 private beds in a clinical unit.

Public beds are divided into four inpatient units: 1) female (five beds for women with disorders related to the use of psychoactive substances and 15 for women with mental disorders); 2) male dependence (20 beds for men with disorders related to the use of psychoactive substances); 3) male disorders (20 beds for men with mental disorders) and 4) residents (31 men and 16 women with chronic mental disorders who do not have family/social support to return to society).

### Population, sample, and selection criteria

The study population is composed of people who were hospitalized in the psychiatric hospital before and after the implementation of the smoking ban. The sample consisted of patients discharged from psychiatric hospitalization between September 2017 and August 2018. Therefore, data referring to 2142 hospitalizations were analyzed.

Inclusion criteria: having been discharged from the hospital between September 2017 and August 2018. Individuals from the residents unit and those discharged from hospitalizations in the clinical unit were excluded.

### Instruments used to collect information

Two instruments were developed specifically for this study, aiming to guide the collection of data from the medical records. These instruments are original and have not been published: 1) “Hospital Indicators and Medication Costs” (ICM) and 2) “Identification of Patients’ Behavior and Care Actions recorded in the medical records (ICR)”.

The ICM is composed of nine hospital indicators (patient-days, number of admissions, occupancy rate, mean length of hospital stay, discharge with improvement, discharge on request, discharge for abandonment of treatment, transfer, and evasion) and seven medications and its cost in reais (oral and intramuscular haloperidol, oral and intramuscular promethazine, oral and intramuscular chlorpromazine, oral and intramuscular diazepam, oral lorazepam, oral clonazepam, and expectorant).

The ICR is composed of eight identification variables (gender, age, psychiatric diagnosis, date of admission, date of discharge, type of hospitalization, type of discharge, tobacco smoking) and eight variables referring to the behavior of patients (verbal aggression, physical aggression and escape and suicide attempts) and to nursing care actions done in coordination with the interdisciplinary team (anticipation of psychotropic drugs, chemical, mechanical and physical restraint). Referrals of patients to Special Care Units (ECU) to keep them away from other patients and in constant supervision of nursing professionals without the use of restraint straps were considered physical restraints. As the present study is ecological, the identification variables were not analyzed.

### Data collection

After approval by the Research Ethics Committee, the IT team and the responsible pharmacy technician were requested to provide the data for the instrument “Hospital Indicators and Medication Costs” (ICM). The data provided referred to all public and private inpatient units, except for the clinical unit.

To fill out the instrument “Identification of Patients’ Behavior and Care Actions recorded in the medical records”, the IT technical team was asked to identify patients discharged from psychiatric hospitalizations in female public units (beds for women with mental disorders and for those with substance use disorders) and male public units (beds for men with mental disorders) between September 2017 and August 2018. Then, based on this list, one of the researchers consulted the electronic medical records referring to hospitalizations that occurred between September 2017 and August 2018. The clinical evolution of patients, the actions carried out by the nurses, and the notes taken by the nursing technicians were read. For each patient from the list provided by the IT team, the researcher filled out the ICR with the information obtained from the medical records. For each variable, the date and time of the event were recorded (for example, verbal aggression: 12/11/2017 at 2 pm) and, in the end, the total number of events was presented. The time was mentioned along with the date as some events occurred more than once on the same day.

### Data treatment and statistical analysis

The Stata/IC (2013) program was used for statistical analysis. The median and interquartile ranges were used as descriptive statistics measures.

The indicators patient-days, number of admissions, occupancy rate, mean length of hospital stay, discharge with improvement, discharge on request, discharge for abandonment of treatment, transfer, and evasion were provided by the hospital’s IT team for each of the 12 months included in the study. For each indicator, two medians and two interquartile ranges were calculated: indicator before the smoking ban (using data from September 2017 to February 2018) and indicator after the smoking ban (using data from March to August 2018). Therefore, median and interquartile ranges were calculated for two six-month periods (six months before the ban and six months after the ban).

To calculate the median and interquartile range of medication costs, the total amount (in reais) that the institution studied spent *per* month on each drug was obtained. The amount spent from September 2017 to February 2018 was considered to generate the indicator “medicine cost before the smoking ban” and the amount spent from March to August 2018 generated the indicator “medicine cost after the smoking ban”. A similar procedure was performed for the indicators verbal aggression, physical aggression, ECU referrals, mechanical restraint, chemical restraint, psychotropic drug anticipation, escape attempts and suicide attempts, using the monthly data obtained from the medical records of the participants included in the study.

For the bivariate analysis, the median test (one-sided Fisher’s exact procedure) was applied to compare the two periods, before and after the smoking ban. For each variable, the *p-value* was calculated in isolation (observed *p-value)* and with Holm-Bonferroni correction for multiple comparisons (corrected *p-value).* The significance level was set at 5%. The discussion was based on the scientific literature on the subject.

### Ethical aspects

The project was registered on the Brazil/CONEP Platform (CAAE 79316817.7.0000.5393) and approved by the Research Ethics Committee of the Ribeirão Preto School of Nursing (EERP/USP nº 307/2017). The Research Ethics Committee was asked to waive the requirement for the Informed Consent Form (ICF), as the data used were secondary.

## Results

There were 2142 psychiatric admissions from September 2017 to August 2018 in the studied hospital. Hospital indicators and data obtained from electronic medical records refer to this universe.

Table 1 shows a comparison of hospital admission considering two periods: before and after the implementation of the smoking ban.

**Table 1:** Comparison of hospitalization indicators, before and after the smoking ban (n= 2142). São Paulo, Brazil, 2018

Hospital indicators	Before ban	After ban	Total	Observed *p-value*	Adjusted *p-value*
	Median (IQ * )	Median (IQ*)	Median (IQ*)		
*Patient-days*					
Private	1814 (176)	1846 (50)	1845 (130)	0.716	0.013
Day Hospital	196 (18)	224 (95)	205 (72)	0.284	0.003
Female	1150 (159)	1124 (63)	1127 (107)	0.716	0.013
Male (disorders)	983 (141)	634 (29)	767 (349)	0.001^†^	0.001^†^
Male (addiction)	1354 (62)	616 (66)	1112 (738)	0.001^†^	0.001^†^
Total	7924 (424)	6378 (309)	7148 (1546)	0.001^†^	0.001*
*Number of admissions*					
Private	54.5 (72)	45.5 (7)	48.5 (23.5)	0.284	0.003
Day Hospital	7.0 (6)	4.5 (3.0)	5.5 (4.5)	0.284	0.003
Female	47.5 (6)	18.5 (7)	42.0 (29)	0.008^†^	0.001
Male (disorders)	28.0 (9)	14.0 (6)	20.5 (14)	0.001^†^	0.001^†^
Male (addiction)	53.5 (10)	29.0 (3.0)	38.0 (24.5)	0.001^†^	0.001^†^
Total	204.5 (79.0)	130.5 (24)	170.5 (74)	0.040^†^	0.001
*Occupancy rate*					
Private	89.6 (8.7)	87.6 (2.4)	87.6 (6.9)	0.716	0.013
Day Hospital	18.2 (44.8)	76.6 (23.5)	62.1 (63.4)	0.040^†^	0.001
Female	75.9 (6.2)	72.9 (2.2)	73.7 (3.4)	0.284	0.003
Male (disorders)	88.8 (10.3)	48.4 (4.4)	69.4 (40.4)	0.001^†^	0.001^†^
Male (addiction)	94.4 (1.4)	42.8 (5.6)	79.7 (51.6)	0.001^†^	0.001^†^
Total	75.8 (7.1)	64.9 (2.8)	72.8 (10.9)	0.040^†^	0.001
*Mean length of stay*					
Private	17.9 (0.9)	17.6 (0.8)	17.6 (1.1)	0.716	0.013
Day Hospital	17.1 (4.3)	19.4 (2.0)	18.7 (3.6)	0.284	0.003
Female	14.7 (0.8)	19.5 (1.1)	15.4 (4.8)	0.040^†^	0.001
Male (disorders)	17.2 (1.5)	16.7 (4.0)	16.9 (2.4)	0.716	0.013
Male (addiction)	13.5 (1.8)	12.6 (0.5)	13.1 (1.4)	0.040^†^	0.001
*Total*	*4.2 (0.2)*	*3.3 (0.2)*	*3.8 (0.8)*	*0.001* ^*†*^	*0.001* ^*†*^

Source: Information system of the study site. *IQ = Interquartile range; ^†^ Evidence of statistical association.

Due to the significant reduction in hospitalizations in some units, Table 1 compares not only the absolute numbers (hospitalizations and patient-days) but also the bed occupancy rate, to allow comparisons between units. The analysis of the hospital admission variables (number of admissions, patient-days, bed occupancy rate and mean length of hospital stay) of the five units considered together (total *per* indicator) showed statistical evidence of difference before and after the implementation of the smoking ban. However, when analyzing each unit, statistical differences are found only in the public units, except the Day Hospital, in which there was an increase in bed occupancy rate after the beginning of the ban (Table 1).

In the two male public units (psychiatric disorders and substance dependence), there was a reduction in the number of hospitalizations, in the number of patient-days and in the bed occupancy rate. This occurred both in the median test with the variables considered in isolation and in the test that considered the entire group of variables. In the female unit, in turn, there was statistical evidence of a reduction in the number of hospitalizations only when the test considered the variables in isolation (Table 1).

The analysis of the five inpatient units together showed statistical evidence of a reduction in the mean length of hospital stay after the implementation of the ban. The separate analysis of the units showed a reduction in the length of stay in male units, with statistical significance only in the substance dependence unit. The female unit, in turn, showed the opposite trend, with an increase in the length of hospital stay (Table 2).

Table 2 shows the comparison of hospital discharge indicators before and after the smoking ban.

**Table 2:** Comparison of hospital discharge indicators before and after the smoking ban (n= 2142). São Paulo, Brazil, 2018

Hospital indicators	Before ban	After ban	Total	Observed *p-value*	Adjusted *p-value*
	Median (IQ*)	Median (IQ*)	Median (IQ*)		
*Discharge with improvement*					
Private	30.0 (5.0)	28.0 (4.0)	29.0 (5.5)	0.121	0.002
Day Hospital	5.0 (3.0)	2.0 (2.0)	3.0 (3.5)	0.121	0.002
Female	27.5 (7.0)	14.5 (3.0)	23.5 (13.0)	0.001^†^	0.001^†^
Male (disorders)	17.0 (8.0)	9.0 (4.0)	11.0 (8.0)	0.121	0.002
Male (addiction)	34.5 (8.0)	18.0 (5.0)	24.5 (16.5)	0.001^†^	0.001^†^
Total	118.5 (6.0)	74.0 (8.0)	89.0 (44.5)	0.001^†^	0.001^†^
*Discharges on request*					
Private	3.0 (3.0)	4.5 (3.0)	3.5 (3.0)	0.284	0.003
Day Hospital	-	-	-	-	-
Female	4.0 (4.0)	1.0 (1.0)	3.0 (3.0)	0.121	0.002
Male (disorders)	0.5 (2.0)	1.5 (2.0)	1.0 (2.0)	0.500	0.005
Male (addiction)	6.5 (8.0)	3.0 (1.0)	3.5 (3.5)	0.284	0.003
Total	16.5 (9.0)	10.5 (3.0)	12.5 (7.0)	0.040^†^	0.001
*Discharges for treatment abandonment*					
Private	1.0 (1.0)	1.0 (1.0)	1.0 (1.0)	0.500	0.005
Day Hospital	-	-	-	-	-
Female	2.5 (2.0)	0.0 (1.0)	1.0 (2.5)	0.008^†^	0.001
Male (disorders)	0.5 (2.0)	0.0 (1.0)	0.0 (1.0)	0.500	0.005
Male (addiction)	1.0 (1.0)	0.0 (0.0)	0.0 (1.0)	0.008^†^	0.001
Total	6.5 (4.0)	1.5 (1.0)	2.0 (5.0)	0.008^†^	0.001
*Transfers*					
Private	2.0 (2.0)	2.0 (2.0)	2.0 (2.0)	0.500	0.005
Day Hospital	0.0 (0.0)	1.0 (1.0)	0.0 (1.0)	0.121	0.002
Female	2.0 (2.0)	0.5 (1.0)	1.0 (1.0)	0.121	0.002
Male (disorders)	0.5 (1.0)	0.0 (1.0)	0.0 (1.0)	0.121	0.002
Male (addiction)	1.0 (1.0)	1.0 (1.0)	1.0 (1.0)	0.500	0.005
Total	5.5 (1.0)	5.0 (5.0)	5.0 (3.0)	0.500	0.005
*Evasion*					
Private	0.0 (0.0)	0.0 (0.0)	0.0 (0.0)	0.500	0.005
Day Hospital	-	-	-	-	-
Female	0.0 (0.0)	1.0 (2.0)	0.0 (1.5)	0.121	0.002
Male (disorders)	2.5 (2.0)	1.0 (2.0)	2.0 (1.5)	0.091	0.002
Male (addiction)	7.0 (3.0)	2.0 (1.0)	5.5 (5.0)	0.001^†^	0.001^†^
*Total*	*11.0 (5.0)*	*3.5 (3.0)*	*8.5 (7.5)*	*0.040* ^*†*^	*0.001*

Source: Information system of the study site. *IQ = Interquartile range; ^†^ Evidence of statistical association.

The analysis of the hospital discharge indicators showed a reduction in the number of discharges with improvement in the five units, which is in accordance with the reduction in the number of hospitalizations when comparing the two periods (Table 2).

Although there is no statistical significance, there was an increase in the number of discharges on demand in the private unit and in the male unit for psychiatric disorders, contrary to the trend of the other units, in which there was a reduction in this type of discharge (Table 2).

There is statistical evidence of a reduction in evasion in the male unit for substance dependence, both in the median test considering the variable isolated and in the test with the set of variables.

Table 3 compares the costs of psychotropic drugs and expectorants in the six months before the ban and the six months after it.

**Table 3:** Comparison of medication costs (in reais) before and after the smoking ban (n= 2142). São Paulo, Brazil, 2018

Medication (administration route)	Before ban	After ban	Total	Observed *p-value*	Adjusted *p-value*
	Median (IQ*)	Median (IQ*)	Median (IQ*)		
Haloperidol (oral)	296.4 (46.0)	228.1 (89.1)	286.1 (74.8)	0.284	0.008
Haloperidol (intramuscular)	65.8 (41.4)	69.8 (37.1)	69.8 (37.9)	0.716	0.025
Promethazine (oral)	159.4 (43.5)	137.8 (8.1)	145.5 (25.4)	0.040^†^	0.005
Promethazine (intramuscular)	124.5 (47.5)	118.5 (70.9)	121.4 (51.0)	0.716	0.25
Chlorpromazine (oral)	746.2 (136.9)	719.7 (83.8)	734.4 (60.2)	0.284	0.008
Chlorpromazine (intramuscular)	9.5 (15.4)	6.0 (4.3)	7.3 (6.1)	0.284	0.008
Diazepam (oral)	110.0 (38.4)	88.4 (24.5)	95.6 (39.3)	0.284	0.008
Diazepam (intramuscular)	1.3 (0.9)	3.1 (2.5)	2.0 (2.8)	0.284	0.008
Lorazepam (oral)	414.9 (65.3)	412.5 (42.4)	412.5 (54.4)	0.716	0.025
Clonazepam (oral)	156.5 (18.7)	128.8 (56.5)	152.1 (42.8)	0.040^†^	0.005
Expectorant (oral)	92.6 (175.8)	73.6 (28.9)	85.7 (39.6)	0.284	0.008

Source: Pharmacy technician responsible for the study site. *IQ = Interquartile range; ^†^ Evidence of statistical association.

In general, it is shown that, after the implementation of the smoking ban, there was a decrease in the costs of psychotropic drugs and expectorants. However, the separate analysis shows evidence of statistical significance only for the variables Promethazine (oral) and Clonazepam (oral).

Table 4 shows the comparison of patients’ behaviors and care actions before and after the ban.

**Table 4:** Comparison of patients’ behavior before and after the smoking ban (n= 2142). São Paulo, Brazil, 2018

Behaviors	Before ban	After ban	Total	Observed *p-value*	Adjusted *p-value*
	Median (IQ*)	Median (IQ*)	Median (IQ*)		
Total verbal aggression	14.5 (5.0)	7.0 (5.0)	10.5 (8.5)	0.040^†^	0.003
Female unit	8.0 (6.0)	4.5 (6.0)	7.5 (5.5)	0.284	0.006
Male units	3.5 (5.0)	1.5 (1.0)	2.0 (3.0)	0.121	0.003
Total physical aggression	14.0 (5.0)	5.5 (4.0)	9.5 (8.5)	0.040^†^	0.003
Female unit	10.0 (3.0)	3.0 (3.0)	7.0 (7.0)	0.040^†^	0.003
Male units	4.0 (2.0)	2.0 (3.0)	3.0 (3.5)	0.273	0.004
Total ECU referrals	19.0 (5.0)	7.0 (1.0)	12.0 (12.0)	0.008^†^	0.002
Female unit	13.0 (5.0)	4.0 (2.0)	6.0 (9.0)	0.008^†^	0.002
Male units	5.5 (3.0)	2.5 (2.0)	4.0 (3.5)	0.121	0.003
Total mechanical restraint	13.0 (3.0)	12.5(12.0)	13.0 (7.0)	0.727	0.005
Female unit	7.0 (2.0)	8.5 (8.0)	7.0 (5.5)	0.500	0.004
Male units	8.0 (4.0)	3.5 (5.0)	5.5 (6.5)	0.284	0.003
Total anticipation of psychotropic drugs	7.0 (5.0)	3.0 (2.0)	4.0 (4.0)	0.030^†^	0.002
Female unit	6.0 (3.0)	2.0 (2.0)	3.5 (4.0)	0.040^†^	0.002
Male units	1.0 (1.0)	0.0 (1.0)	1.0 (1.0)	0.227	0.002
Total escape attempts	1.5 (2.0)	1.0 (1.0)	1.0 (1.5)	0.273	0.003
Female unit	1.0 (0.0)	1.0 (1.0)	1.0 (0.5)	0.500	0.003
Male units	0.0 (1.0)	0.0 (0.0)	0.0 (0.5)	0.500	0.003
Total suicide attempts	0.0 (0.0)	0.0 (1.0)	0.0 (0.5)	0.500	0.003
Female unit	0.0 (0.0)	0.0 (0.0)	0.0 (0.0)	0.500	0.003
Male units	0.0 (0.0)	0.0 (1.0)	0.0 (0.0)	0.227	0.002
Total chemical restraint	58.5 (10.0)	37.5 (10.0)	50.0 (21.0)	0.001^†^	0.002^†^
Female unit	46.0 (12.0)	31.0 (6.0)	38.5 (15.0)	0.040^†^	0.002
Male units	14.5 (3.0)	5.0 (4.0)	9.5 (9.5)	0.040^†^	0.002

Source: Consultation of electronic medical records. *IQ = Interquartile range; ^†^Evidence of statistical association.

Despite the absence of statistical difference in the number of mechanical restraints when comparing the periods before and after the smoking ban, there is statistical evidence of a decrease in episodes of verbal and physical aggression, referrals to the ECU, anticipation of psychotropic drugs, and chemical restraints. No differences were observed in escape and suicide attempts.

## Discussion

To our knowledge, this is the first Brazilian study to analyze a psychiatric hospital by comparing hospital indicators, medications costs, and behavior of inpatients before and after the implementation of a smoking ban. The study results are important as they show the benefits of the smoking ban for psychiatric patients, which can encourage nurses and other professionals to contribute to the implementation of smoking bans in mental health services and help them understand the relevance of their role when thinking about smoking cessation after hospital discharge.

The hypothesis that the smoking ban affects hospital indicators was confirmed. After the ban, there was a reduction in the number of patient-days, in the number of admissions, in bed occupancy rate, and in the length of hospital stay. However, when analyzing the indicators per unit, it is shown that the reductions occurred mainly in public inpatient units, especially male ones.

The reduction of bed occupancy rate and length of hospital stay in public male units, but not in the private unit, contradicts the arguments of nursing professionals from the latter, who defend that the smoking ban not should continue, as it would affect the number of admissions and, consequently, the hospital budget^7^.

From these results, the question that arises is: what can explain the reduction in bed occupancy rates only in male units? It can be inferred that this occurred due to the higher prevalence of smokers among men, as evidenced in other studies^2^ ^,^ ^4^ ^,^ ^12^.

As for the length of hospital stay, the only unit with a reduction in this variable was the one intended for the hospitalization of men with disorders related to the use of psychoactive substances (alcohol and illicit drugs). This result is in line with an American study (n=255) carried out in a psychiatric hospital with a smoking ban, which found that people with substance use disorders have shorter lengths of hospital stay when compared to those with other psychiatric diagnoses^8^.

As shown in American studies, due to the high prevalence of smokers among users of alcohol and other drugs, people with substance use disorders may see cigarettes as an alternative to compensate for the withdrawal from other substances during psychiatric hospitalization. In this sense, the prohibition of smoking in mental health services can be difficult for this public^13^ ^-^ ^14^.

A study carried out in the United Kingdom analyzed 4,223 psychiatric hospitalizations and found results that contradict those of the present study regarding the reduction of the length of hospital stay after the implementation of the smoking ban^15^. These differences in results are understandable, since the studies are carried out in countries with different cultures and with different times and approaches to anti-smoking laws.

Despite the reduction in the length of hospital stay in the male unit for substance use disorders, there was also a reduction in discharges on request and evasion in this unit. This suggests that hospital discharge decisions were made by the interdisciplinary team and not by the inpatients. These results are important, as the fear of an increase in discharges on request and evasion is one of the main arguments of professionals who are against the smoking ban. This was evidenced in the statements obtained in a qualitative Brazilian study and in two quantitative studies carried out with professionals from Australia and Qatar, in which 93% and 73% of professionals, respectively, used this argument to defend their point of view regarding smoking bans^7^ ^,^ ^16^ ^-^ ^17^.

This reduction in discharges on request and evasion after the implementation of the smoking ban still raises the question: did users of alcohol and other drugs get some kind of privilege in the psychiatric hospital after the implementation of the smoking ban?

Qualitative research carried out in the same setting as the present study showed that, after the implementation of the smoking ban, inpatients started exchanging belongings (clothes, hygiene products, etc.) and sexual favors for hidden cigarettes^7^. The area where the unit for substance users is located allows them to access the outside world (by jumping over the wall) and return to the hospital before the nursing team notices their absence. These outings could help them obtain cigarettes outside the hospital and then sell them to people hospitalized in other units.

Likewise, a British study revealed the existence of a trade of objects used for smoking during psychiatric hospitalization, since fights between patients were preceded by theft or disagreement during sales/exchanges of cigarettes and lighters^18^.

Regarding spending on psychotropic drugs and expectorants, the hypothesis that smoking bans favor less spending on these items was confirmed. Although no statistical difference was evidenced, which can be understood by the reduced number of months compared (six months before and six months after the implementation of the ban), it is noted that expenditure on expectorants had an average reduction of 20%.

A Spanish study carried out in a psychiatric inpatient service with 276 patients showed that 48% of smokers presented with cough, 41% with expectoration, and 36% had a diagnosis of chronic bronchitis. Among non-smokers, the prevalence of these conditions was less than 3%^19^. This result is an example of the physical harm caused by smoking, which is widely reported in the scientific literature and in publications of government bodies^2^ ^,^ ^9^. Therefore, it is expected that the smoking ban in mental health services leads to a reduction in expenses with expectorants, as smoking cessation is associated with improvement in physical health, including respiratory symptoms.

Scientific evidence obtained in American and Italian studies addressing the physical harm caused by smoking among people with mental disorders shows that smoking is one of the factors associated with the reduction in the life expectancy of this population and the high prevalence of somatic comorbidities^20^ ^-^ ^21^. During the COVID-19 pandemic, European authors revived this discussion, expressing that smoking is an important risk factor for COVID-19 complications in this population^22^.

As for psychotropic medication costs, the amount spent on the antipsychotics oral haloperidol and intramuscular chlorpromazine was reduced by 23% and 37%, respectively, when comparing their use before and after the beginning of the ban. Cost reduction was also observed in the anxiolytics oral clonazepam (18%) and diazepam (20%).

These results are in agreement with a British study carried out with 13,846 people with mental disorders, which showed an association between increased medication costs and smoking^10^. Although cost reduction is an important argument used by nurses to defend the smoking ban in mental health services, the most important aspect of this phenomenon is the reason for this reduction: the improvement of psychiatric symptoms and, consequently, the need for lower dosis of medication.

Considering that knowledge of hospital indicators and medication costs is important for daily decision-making in mental health services, the changes in these variables after the smoking ban show that the anti-smoking law has the potential to positively impact the management processes of these services. This way, nurses in possession of this knowledge can have a more positive attitude towards the implementation of the law, as they know that the benefits of the smoking ban are compatible with the expectation of improving the mental and physical health of people with mental disorders and with the health funding available. Thus, the cost-benefit ratio is unquestionable.

As described in the scientific literature, smoking exacerbates positive psychotic symptoms (delusions and hallucinations, for example), anxiety, and depression. For this reason, smokers usually receive higher dosis of psychotropic drugs when compared to non-smokers. Furthermore, tobacco increases the metabolism of psychotropic drugs, reducing their concentration in the bloodstream and consequently requiring higher dosis to reach the therapeutic effect^23^ ^-^ ^25^. This scenario explains the higher costs with psychotropic drugs when smoking is allowed in a mental health service, and the decrease in costs when smoking is prohibited.

The improvement in psychiatric symptoms as one of the possibilities for the reduction in psychotropic medication costs is consistent with the confirmation of the third hypothesis of the study, the decrease in episodes of aggression after the implementation of the smoking ban, as evidenced by the decrease in episodes of verbal and physical aggression, in anticipation of psychotropic drugs and in physical and chemical restraints.

Studies conducted in Australia and Qatar show that the main argument against the smoking ban is the belief that patients will become more agitated and aggressive towards the team due to nicotine withdrawal (nine out of 10 professionals have these fears)^16^ ^-^ ^17^. The confirmation of the hypothesis of a decrease in episodes of aggression with the implementation of the smoking ban provides answers to the concerns of the professionals. It is interesting to note that studies carried out in Qatar and England show that professionals who received training in the approach to smoking in the psychiatric population were more in favor of the smoking ban than those who did not, demonstrating the potential of continuing education^16^ ^,^ ^26^.

A British study carried out in 12 psychiatric inpatient wards found that, after the implementation of smokefree policies, there was a decrease in the frequency of incidents such as physical violence against staff (from 58.4% to 20%) and verbal aggression (from 25% to 20%). However, conflicts between patients due to the concealment of cigarettes and negotiations between them increased from 2% to 10%^27^. Likewise, a second British study revealed a 39% reduction in physical aggression after the implementation of the smoking ban, even after controlling for confounding variables (gender, age, psychotic disorders, and judicial hospitalization)^28^. Two systematic literature reviews confirmed that smoking bans are not associated with increased physical and verbal aggression in mental health services. These reviews included studies carried out in Australia, Canada, the United States, and England^29^ ^-^ ^30^.

A review of the scientific literature published in Cochrane found no evidence that smoking cessation worsens the mental health of psychiatric patients. In addition, there is evidence that nicotine withdrawal is associated with an improvement in anxiety and depression^31^.

Evidence of improved behavior after the implementation of the smoking ban offers a new perspective to professionals working in mental health services, as the main arguments against smoking bans in these services are related to fear of worsening the psychiatric condition of inpatients. The positive change in the behavior of patients after the implementation of the smoking ban should be widely publicized, as this knowledge can contribute to improving interpersonal relationships in mental health services. In this sense, as advocated by Australian researchers, psychiatric hospitalization can be seen as an opportunity to initiate a dialogue on smoking cessation^32^.

Study limitations: 1) The period analyzed (six months before the ban and six months after) may not have been sufficient to unveil all the differences in hospital indicators, patient behavior, and medications costs; 2) There is the possibility of information bias, as some data (aggressions and escape attempts, for example) may not have been recorded in the medical records; 3) There is a risk of ecological fallacy in the belief that associations based on aggregate data can be applied to individuals.

Implications for the advancement of scientific knowledge in nursing: By comparing hospital indicators, drug costs, and patient behavior before and after the implementation of the smoking ban, the present study offers nurses a new perspective on the anti-smoking law. By providing scientific evidence that the implementation of the ban is associated with a reduction in verbal and physical aggression and in physical and chemical restraints, the present study contradicts the myth that the smoking ban leads to an increase in aggressive behavior. Since nurses spend the most time with hospitalized patients, understanding the health benefits of smoking cessation for hospitalized smokers allows a reflection on nursing practice and on strategies to deal with the resulting problems. If nurses review their beliefs there can be positive results both for interpersonal relationships and for decision-making in management processes in mental health services.

## Conclusion

The implementation of the smoking ban resulted in changes in hospital indicators in all inpatient units, except for the private inpatient unit. In addition, the ban resulted in a reduction in costs on psychotropic drugs and expectorants, and a decrease in episodes of verbal and physical aggression.

It is hoped that the present study will help nurses to review beliefs and myths related to the prohibition of smoking in mental health services, as there is evidence of positive changes for the management of services and of improvement in the behavior of people with mental disorders after the implementation of this measure.
